# Posttraumatic Stress Disorder: An Immunological Disorder?

**DOI:** 10.3389/fpsyt.2017.00222

**Published:** 2017-11-06

**Authors:** Zhewu Wang, Blaine Caughron, M. Rita I. Young

**Affiliations:** ^1^Mental Health Service, Ralph H. Johnson VA Medical Center, Charleston, SC, United States; ^2^Department of Psychiatry, Medical University of South Carolina, Charleston, SC, United States; ^3^Research Service, Ralph H. Johnson VA Medical Center, Charleston, SC, United States; ^4^Department of Otolaryngology – Head and Neck Surgery, Medical University of South Carolina, Charleston, SC, United States

**Keywords:** Immune, Inflammation, PTSD, Stress, Disorder

## Abstract

Patients with posttraumatic stress disorder (PTSD) exhibit an increased state of inflammation. Various animal models for PTSD have shown some of the same immune imbalances as have been shown in human subjects with PTSD, and some of these studies are discussed in this review. However, animal studies can only indirectly implicate immune involvement in PTSD in humans. This review of mainly studies with human subjects focuses on dissecting the immunological role in the pathogenesis of PTSD following initial trauma exposure. It addresses both the inflammatory state associated with PTSD and the immune imbalance between stimulatory and inhibitory immune mediators, as well as variables that can lead to discrepancies between analyses. The concept of immunological treatment approaches is proposed for PTSD, as new treatments are needed for this devastating disorder that is affecting unprecedented numbers of Veterans from the long-standing wars in the Middle East and which affects civilians following severe trauma.

## Immune Imbalances in Posttraumatic Stress Disorder (PTSD)

Posttraumatic stress disorder is a debilitating psychiatric disorder that follows trauma exposure. There are four symptom clusters that characterize PTSD: reliving the traumatic event, avoidance of situations reminiscent of the traumatic event, negative thoughts and mood, and hyperarousal. These symptoms are debilitating to function. Trauma exposure is a required risk factor for developing PTSD, but is not sufficient as not all who are exposed to trauma develop PTSD (1, 2). The complex phenotype of PTSD emerges from interactions among genetic, environmental, and other biological risk factors. Dissecting the causes of PTSD could identify individuals who would be at increased risk of developing PTSD following trauma exposure.

A number of studies assessing cytokine levels and, in a few instances, blood immune cell functions have provided support for immunological involvement in PTSD following an initial trauma event. Although somewhat inconsistent, the compilation of these studies points to immune alterations in PTSD that indicate the immunological balance is skewed toward a pro-inflammatory state (Table 1). This is supported by increased levels of pro-inflammatory cytokines such as IFN-γ, IL-6, TNF-α, and IL-17 in the plasma, and increased levels of immune stimulatory Th1 and inflammatory Th17 cells in the blood (3–8). The increase in levels of the pro-inflammatory mediators IL-12 and IFN-γ in plasma of PTSD subjects is associated with multiple genetic and epigenetic modifications in peripheral blood mononuclear cells (9).

**Table 1:** Correlative association between posttraumatic stress disorder and immune imbalance in humans.

	Source	Mediator	Reference	Interpretation caution
Increase in inflammatory cytokine or cell levels	Plasma	IL-2 IFN-γ IL-6 TNF-α IL-12 IL-17	(5–9)	Depression associated with increase in pro-inflammatory cytokines Diurnal variations May be influenced by type of trauma, time since trauma

	Saliva	IL-2 IFN-γ IL-6 IL-17 CCL2	(6, 25, 26)	Oral health conditions increase pro-inflammatory cytokines

	Blood cell secretion	IL-1 IL-6 TNF-α	(22)	Leukocyte cytokine secretion may not reflect plasma levels

	Blood cells	Th1 Th17	(3)	

Decrease in inhibitory cytokine or cell levels	Plasma	TGF-β IL-4 IL-10	(5, 6, 8, 20, 21)	Decrease may be difficult to see if normal levels are low

	Saliva	IL-4 IL-10	(6)	

	Blood cells	Treg	(3, 23)	

Multiple molecular genetics studies that have sought to pinpoint genetic variations associated with the risk for PTSD after trauma exposure (10). Some of these include genes encoding mediators involved in immune regulation. The best studied is the FK506-binding protein 5 gene, FKBP5, which encodes an immune regulatory immunophilin (11). FKBP5 gene variants moderate the effect of trauma exposure on the risk of PTSD. The minor allele of FKBP5 SNPs rs1360780 was associated with increased hurricane-associated PTSD (12). In subjects with chronic pain, heterozygous T-allele carriers of the FKBP5 SNP rs9470080 were associated with an increased risk for PTSD (13). A study of mainly African-American subjects who had experienced severe childhood trauma showed that those who carried minor alleles of FKBP5 SNPs rs9296158, rs3800373, rs1360780, or rs9470080 were more likely to exhibit PTSD (14).

In addition to FKBP5 polymorphisms, there are associations between variants in the pro-inflammatory C-reactive protein (CRP) and PTSD. A study of mainly African-American inner-city individuals who had been exposed to trauma showed a link between a CRP SNP, rs1130864, and the risk of PTSD, with the most prominent symptom of being overly alert (15). This risk SNP was also associated with increased serum CRP levels, which indicates an inflammatory state.

Multiple studies have indicated that immune hyperactivation could be a predictor of PTSD risk. For example, high levels of CRP in Marines before their deployment were predictive of PTSD following deployment; this study suggested inflammation to be a contributor to PTSD (16). Blood levels of inflammatory cytokines were increased in hospitalized patients with traumatic orthopedic injuries who subsequently developed PTSD (8). In a pre- and post-deployment analysis that used whole-transcriptome RNA-Seq gene expression of blood from U.S. Marines, both pairs of samples from subjects who developed PTSD over-expressed genes enriched for immune responses (17). This supports the concept that immune hyperactivation before trauma exposure could predict PTSD.

Some studies have shown that increases in inflammatory mediators correlate with PTSD severity (3), although others have not (18). A polymorphism in the gene of the inflammatory marker, TNF-α (rs1800629) was associated with PTSD in Vietnam war combat Veterans, and correlated with PTSD severity (19). While rs1800629 was a risk genotype for PTSD severity, serum levels of TNF-α were associated with symptom severity, but only trended to significance when controlling for covariates.

Although studies have shown increases in pro-inflammatory mediators in subjects with PTSD, fewer have measured inhibitory cytokines (5, 8, 20). In such studies, PTSD subjects have generally lower inhibitory cell levels such as Treg and reduced levels of the inhibitory mediators TGF-β and IL-4 (3, 6) in the blood. A comparison of individuals who were exposed to urban violence-associated trauma showed that those with PTSD had lower blood levels of the inhibitory mediator IL-10 than those who were resilient to the trauma (21). The importance of these deficiencies in immune inhibitory regulators is that a healthy immune status is composed of a balance of stimulatory and inhibitory cells and mediators. In studies where balances in stimulatory and inhibitory mediators were assessed, immune skewing was toward the pro-inflammatory direction in Veterans and civilians with PTSD (6, 8).

Most studies examining the changes in cytokine levels in PTSD have measured cytokine levels in the blood. However, several studies have also examined the sources of the pro-inflammatory cytokines. Blood leukocytes of war-exposed refugees with PTSD spontaneously produced increased levels of IL-1, IL-6, and TNF-α than did leukocytes of controls, although plasma cytokine levels were similar among the two groups of subjects (22). Also shown in this study was a direct correlation between PTSD severity and spontaneous secretion of IL-6 and TNF-α by the leukocytes. Stimulation of the PTSD subjects’ blood leukocytes with LPS further increased IL-6 production to higher levels than those produced by leukocytes of controls. A separate analysis of peripheral blood of combat Veterans with PTSD showed increases in blood pro-inflammatory Th1 and Th17 subsets and a reduction in the number of inhibitory Treg (3). Reduced levels of Treg cells were similarly seen in war-exposed civilians with PTSD as compared to exposed civilians without PTSD or controls (23). In contrast, a separate study comparing war Veterans to age-matched healthy controls showed reduced T-cell production of pro-inflammatory cytokines IL-2 and IFN-γ (24). Possible reasons for the discrepancies among some of these studies are discussed below.

There have been a few studies that also examined levels of immune mediators in saliva. Similar to the blood observations, inflammatory mediator levels in saliva were increased following stress exposure (25, 26). Veterans with PTSD had higher levels of pro-inflammatory mediators IL-2, IFN-γ, IL-6, and IL-17 and reduced levels of the inhibitory mediators IL-4 and IL-10 in saliva compared to Veterans without combat-related PTSD (6). In this latter study, it was shown that the immune cytokine imbalances in PTSD patients are more prominently expressed in saliva than in blood (6). A study of hurricane survivors with PTSD showed that the increased saliva levels of the inflammatory mediator CCL2 (MCP-1) correlated with PTSD severity (25). The origin of cytokines in saliva and whether salivary cytokine levels are a reflection of blood levels has been questioned, but increases in inflammatory cytokines have appeared rapidly in saliva following acute stress and could reflect mental health status (26–28).

Comorbidities need to be considered when studying immune imbalances in saliva, plasma, or blood cells of PTSD subjects. For example, oral health increases salivary cytokine levels in individuals with periodontitis, gingivitis, premalignant oral lesions, and oral cancer (29, 30). Depression, which is common in subjects with PTSD, also increases levels of inflammatory cytokines (31). However, several studies that examined the contribution of depression to the increases in inflammatory cytokines in subjects with PTSD showed that such increases were independent of a depression diagnosis (6, 32). Other variables that can impact on cytokine or immune cell analyses could include treatments and recovery from PTSD. Traumatized women with PTSD had increased plasma levels of IL-6, but those who had recovered from PTSD had the same lower levels as did healthy controls (33). The reduced levels of Treg in war-exposed PTSD subjects were restored to levels of healthy controls following narrative exposure therapy (23). Cytokine levels can also be influenced by technical complications. For example, cytokines such as IL-1 and IL-6 exhibit diurnal variations, which can contribute to differing results among studies (34, 35). The type of trauma can also impact on cytokine measurements. This was highlighted by an analysis showing that interpersonal-related traumas had distinct gene expression signatures from combat-related traumas, but there was convergence on immune cascades between the different trauma categories (36).

More difficult to control in studies with human subjects is the impact of the duration between the PTSD-associated trauma and the time of immunological analysis, in particular if the immunological skewing is a predisposing factor for PTSD (37). However, this can be controlled in animal models of PTSD. Using the stress-enhanced fear learning model, IL-1 expression was shown to rapidly increase (within 6 h) in the dorsal hippocampus and remain increased for the 72-h duration of assessments (38). Brain levels of IL-α, IL-6, and TNF-α were also increased within 1 day of stress re-exposure in a rat predator stress model for PTSD (39). Also using the predator model, a separate study showed increased brain levels of the inflammatory mediator IL-1 and reduced levels of the inhibitory mediators IL-4 and IL-10 after 7 days following re-exposure (40). While these studies in animal models of PTSD demonstrate dysregulation of cytokines within the brain within a short period of time following stress exposure, there is a deficiency in studies to demonstrate the duration of this immune imbalance. This leaves a gap in understanding the kinetics of the immune imbalances associated with PTSD as diagnosis, immune analysis, and treatment for humans with PTSD typically occur considerably later after exposure to the traumatic event. However, these animal models are in a unique position to determine whether immunological dysregulation is a consequence or a cause of PTSD; this is further discussed below.

## Clinical Impacts of Immune Imbalances in PTSD

Associated with PTSD is not only inflammation but its consequence on poorer health outcomes. For example, health-related quality of life was lower in military persons with PTSD who had higher plasma levels of IL-6 (41). The pro-inflammatory milieu of subjects with PTSD may predispose them to autoimmune diseases since combat Veterans with PTSD have an increased incidence of thyroiditis, inflammatory bowel disease, multiple sclerosis, rheumatoid arthritis, and systemic lupus erythematosus (42). Patients exhibiting trauma-related symptoms had a slower neutrophil recovery time following stem cell transplantation than those without PTSD (43). Other comorbidities of subjects with PTSD include an increased risk of coronary heart disease (25, 44). A study of a Vietnam Era Twin Registry showed that the rate of coronary heart disease in twins with PTSD was twice that of twins without PTSD (44). The increased risk of coronary heart disease in PTSD subjects could be due to the increased levels of immune chemokines such as CCL-2, which recruits monocytes toward the inflamed endothelium (25). Monocytes, whose role has not been extensively examined in PTSD, have upregulation of target genes of the pro-inflammatory NF-κB/Rel family of transcription factors, which further contributes to the inflammatory state of PTD subjects (45).

It could also be argued that the increase in autoimmune diseases and other immune-associated comorbidities of PTSD are not the result of PTSD but are predisposing factors for developing PTSD following a traumatic event. Although the immune skewing in PTSD subjects toward an inflammatory state is becoming established, it is difficult to design studies with human subjects to test causality between immune dysregulation and PTSD. However, triggering of CNS neuroinflammation by peripheral inflammation has been shown in animal models (38, 46–55). Activation of the peripheral immune system by endotoxin injection triggered neuroinflammation in the brain (49, 50). Studies with animal models have also shown the requirement for functional immune competence for anxiety behavior to be evident following stress sensitization. For example, neutralization of the inflammatory cytokine IL-1 lessened the maladaptation to acute psychological stress (38). Also shown was monocyte recruitment from the periphery to the brain in stress-sensitized mice, but splenectomy before stress sensitization prevented monocyte migration to the brain and, in turn, prevented anxiety is stress-sensitized mice (38, 46–55).

Studies with human subjects have been less frequent and less definitive, but nevertheless suggest the contribution of peripheral inflammation to neuroinflammation and behavior (Table 2). Traumatic brain injury resulting from military deployment is associated with inflammation and, more recently, this has been associated with a higher extent of PTSD comorbidity (56). This study speculated that the chronic inflammatory state associated with the traumatic brain injury results in elevated cytokine levels in the central nervous system and, in turn, microglial over-activation. In a clinical trial, patients with acute respiratory distress who were treated with GM-CSF had more severe PTSD symptoms than those treated with placebo (57). Since GM-CSF stimulates proliferation and differentiation of hematopoietic cells and can cross the blood–brain barrier, this study suggested the possibility of GM-CSF stimulating either brain microglia or production of inflammatory cytokines within the brain to result in more severe PTSD.

**Table 2:** Suggestions of causal associations between immune imbalances and posttraumatic stress disorder (PTSD).

	Assessment	Result	Reference

Human studies			
Endotoxin administration to healthy volunteers	Plasma	↑ TNF-α ↑ IL-6 ↑ IL-8	(58)
	CNS	Brain microglial activation	(58)

Immune stimulatory cytokine administration to cancer patients	Plasma	↓ Neurotransmitter precursors including tryptophan	(66)
	PTSD symptoms	↑ Hypervigilance, irritability, anxiety	(66)

**Animal models**			

Peripheral immune activation	Brain	↑ Brain neuroinflammation	(49, 50)
Blocking IL-1 signaling	PTSD symptoms	↓ Symptoms after predator stress	(38)
Blocking monocyte migration to brain	PTSD symptoms	↓ Anxiety in repeated social defeat model	(46, 51)

While not directly studied, it could be expected that increased persistent infection could stimulate a chronic inflammatory state to, in turn, increase vulnerability to PTSD following trauma. This concept could be in part supported by studies with human subjects in which healthy volunteers who systemically received endotoxin exhibited stimulated peripheral levels of inflammatory mediators TNF-α, IL-6, and IL-8, and microglial activation in the brain (58). Once activated, brain microglia release mediators such as nitric oxide, IL-1, IL-6, TNF-α, and glutamate, which impact neurotransmission, neuronal apoptosis, neuroendocrine function, neural plasticity, and behavior (59–64). Induction of peripheral inflammation in healthy human volunteers *via* typhoid vaccination resulted in functional impairments in the form of reduced spatial memory performance (65). The impact of peripheral immune activation on behavior was inadvertently made evident by immune-activating treatment of cancer patients who resulted in symptoms characteristic of PTSD such as hypervigilance, irritability, anxiety, and decreased concentrations of tryptophan, a precursor to the neurotransmitter serotonin (66). The serotonergic axis influences mood, aggression, arousal, anxiety, sleep, learning, nociception, fear, and appetite (67).

## PTSD Treatments in the Context of Immune Imbalances

A few studies have examined the impact of therapies for PTSD on the inflammatory status of patients with PTSD, although more of these studies have been conducted in mouse PTSD models. A study with PTSD patients showed that narrative exposure psychotherapy improved PTSD symptoms and restored the levels of immune inhibitory Treg cells, which were reduced before treatment (23). However, the immune imbalances were not fully corrected as the proportion of naïve T-cell remained low relative to memory T-cells, suggesting premature immune senescence. PTSD subjects who received pharmacotherapy with selective serotonin reuptake inhibitors (SSRIs) improved clinically and showed reductions in levels of the pro-inflammatory mediator IL-1 (68). In a mouse model of PTSD, treatment with the SSRI inhibitor fluoxetine prevented stress-induced inflammatory gene expression and improved PTSD-like symptoms (69). The results of this study indicated the role of inflammation in PTSD pathology and suggested using anti-inflammatory agents to treat PTSD. A rat study of PTSD showed that treatment with ibuprofen to directly target inflammation reduced both levels of inflammatory cytokines and PTSD-like symptoms (70). Similarly, treating mice with intraperitoneal injections of COX-2 inhibitors to diminish inflammation attenuated their PTSD-like symptoms and reduced neuronal excitability in the basolateral amygdala (71).

While studies showing immune restoration with PTSD treatment suggest a psycho-neuro-immune relationship, it is not possible to determine if treatment impacted the PTSD disorder to, in turn, lessen the extent of inflammation, or if the restoration of immune balance led to PTSD psychological improvement. SSRIs, by modulating serotonin levels, may be influencing immune function since serotonin has been shown to be an immune modulator (72–74). There remains a need for studies to determine the associations between improvements in PTSD clinical status and the immune rebalancing.

Despite the availability of psychological and pharmacological treatments for PTSD, approximately half of combat Veterans do not respond favorably to treatments (75). Therefore, studies need to be expanded to assess if PTSD treatment responsiveness or resistance is associated with a respective immune rebalancing or resistance to rebalancing. If the pro-inflammatory state contributes to PTSD, and if inflammation-skewed leukocytes of PTSD subjects still retain plasticity, then there is the opportunity for immune redirection as an additional PTSD treatment approach. Such immune rebalancing approaches have become more common in treating inflammation-associated diseases, such as rheumatoid arthritis or type 1 diabetes, and as treatments for cancer (76–79).

The plasticity of immune T-cells and monocytes provides optimism that the inflammation-skewed immune state in patients with PTSD can be rebalanced. This rebalancing could be driven by the composition of the cytokine milieu (80, 81). Inflammatory Th17 cells share a common lineage with inhibitory Treg cells, and their plasticity is evident by examples of one phenotype differentiating from the other (82). Although cytokines have been widely used to define immune plasticity, a pharmaco-immunological approach may be a more practical means by which to attain immune rebalancing. For example, the STAT3 inhibitor STA-21 was effective in restoring immune balance in a mouse model of inflammatory arthritis (80). Studies in both mouse models and in humans have shown vitamin D metabolites can restore immune balance in several different clinical conditions (83–86). These alternative agents could be used in future clinical studies to restore immune balance in Veterans and civilians, with the goal of tempering the clinical course of their PTSD.

## Conclusion

The interconnections between immune imbalances and PTSD are becoming better defined, but much is left to be resolved. It is clear that PTSD is associated with a pro-inflammatory state but whether this contributes to the symptoms of PTSD or whether it is a consequence of disease has yet to be clarified. There is, however, increasing evidence that hyperinflammation is not only a biomarker for PTSD but increases the risk of PTSD following trauma. This is important to enable identification of those at risk for PTSD that could, for example, result from military combat exposure. It could also expose new opportunities for prevention and treatment of PTSD. Immune modulating approaches are accepted means of treating various disorders such as autoimmune diseases. Unraveling the psycho-neuro-immunological interplay in PTSD is an ongoing challenge that could results in effective means to prevent and to treat PTSD.

## Author Contributions

This manuscript was written by MY with close collaboration and contribution by ZW and BC.

## Conflict of Interest Statement

The authors declare that the research was conducted in the absence of any commercial or financial relationships that could be construed as a potential conflict of interest.
